# Plant Metabolome Between Root and Aerial Parts of *Cichorium intybus* L. and Anti-Hyperuricemia Mechanisms Based on Cell Metabolomics

**DOI:** 10.3390/metabo15110727

**Published:** 2025-11-06

**Authors:** Jingbo Wang, Shi Shen, Qi Zhao, Xin Shen, Qin Zhuo

**Affiliations:** NHC Key Laboratory of Public Nutrition and Health, National Institute for Nutrition and Health, Chinese Center for Disease Control and Prevention, Beijing 100050, China; wangjb@ninh.chinacdc.cn (J.W.); zhuoqin@ninh.chinacdc.cn (Q.Z.)

**Keywords:** *Cichorium intybus* L., chicory, anti-hyperuricemia, plant metabolomics, cell metabolomics, biomarkers

## Abstract

**Background/Objectives**: Hyperuricemia (HUA) is a metabolic disease with increasing incidence. Chicory (*Cichorium intybus* L.), a traditional medicinal and edible plant, has demonstrated anti-HUA effects. However, the metabolic profiles of its aerial parts and roots are still not fully characterized. Moreover, few studies have investigated its anti-HUA effects using cell metabolomics. **Methods**: The metabolomes of chicory root and aerial parts were characterized using UPLC-QTOF-MS-based untargeted metabolomics. Subsequently, the anti-HUA mechanism of chicory root was investigated by performing non-targeted metabolomics in HK-2 cells. **Results**: The results demonstrated that various hydroxycinnamic acids and flavonoids were more abundant in aerial parts, whereas sesquiterpenes and oligosaccharides were characteristic of the root. Both chicory root and aerial part extracts significantly reduced uric acid (UA) levels in HK-2 cells induced by adenosine with xanthine oxidase (XO). Cellular metabolomic profiling indicated a distinct separation between the root extract (CR40, 40 mg/mL) and the model group. OPLS-DA identified 165 differential metabolites, including acylcarnitines, acylamino acids, peptides, phospholipids, glycerides, and lipid-like molecules. These metabolites were associated with key metabolic pathways of sphingolipids, glycerophospholipids, phosphonate and phosphinate, linoleic acid, biotin, purine, as well as taurine and hypotaurine metabolism. **Conclusions**: Chicory is rich in diverse bioactive compounds and exhibits significant anti-HUA activity by modulating multiple metabolic pathways.

## 1. Introduction

With the continuous improvement of living standards, the prevalence of hyperuricemia (HUA) has been increasing annually, making it one of the most common metabolic diseases. In the past decade, the prevalence of HUA in China has increased, with an incidence ranging from 13.3% to 18.4%, affecting approximately 177 million patients [1,2]. HUA is a metabolic disorder characterized by elevated levels of uric acid (UA) due to purine metabolism dysfunction. HUA is closely associated with the cause and progression of various diseases, including gouty arthritis, diabetic nephropathy, hypertension, cardiovascular diseases, dyslipidemia, and non-alcoholic fatty liver disease [3,4]. At present, the drug allopurinol, which inhibits UA synthesis, and the drug benzbromarone, which promotes UA excretion, are used to treat HUA [5]; however, these drugs have achieved success in lowering UA levels but not long-lasting, accompanied by adverse reactions such as rash and gastrointestinal reactions, hypersensitivity and cardiovascular risks, and liver and kidney damage [6,7,8]. Interest in the development of functional foods has increased substantially in recent years across the commercial, academic, and governmental sectors. Many scholars have found that the effective ingredients from natural food have UA-lowering effects, such as celery seed [9], chicory [10], and dandelion [11].

Chicory (*Cichorium intybus* L.), a perennial herb belonging to the genus *Cichorium* in the *Asteraceae* family, is cultivated worldwide. Originating in Europe (particularly the Mediterranean region), it may also be cultivated in all other temperate regions and semi-arid areas [12]. While the roots of chicory are primarily utilized for the extraction of inulin-type prebiotics, the leaves are often consumed fresh and are rich in diverse phytochemicals. A comprehensive review highlights chicory as a promising source of bioactive compounds for food fortification, containing constituents such as inulin, sesquiterpene lactones, caffeic acid derivatives, proteins, lipids, flavonoids, hydroxycoumarins, alkaloids, steroids, terpenoids, essential oils, volatile compounds, vitamins, β-carotene, zeaxanthin, and minerals [13]. These components contribute to a wide range of biological activities, including hepatoprotective, anti-inflammatory, antioxidant, sedative, immunomodulatory, cardiovascular, hypolipidemic, antidiabetic, anticancer, gastroprotective, and antimicrobial effects [14]. In recent years, it has been reported that chicory has a strong anti-HUA effect by suppressing the LPS/TLR4 axis inflammatory reaction in quail model [15]. It was also detected that chicory inulin decreased serum uric acid (UA), triglyceride (TG), and abdominal fat deposition in a quail model of HUA by altering the acetyl-CoA carboxylase (ACC) protein expression and xanthine oxidase (XOD) and fatty acid synthase (FAS) activities [16].

Metabolomics has emerged as a powerful analytical platform for both biomarker discovery and phenotypic characterization, enabling the comprehensive identification of metabolites that modulate cellular and organismal functions [17]. Although previous studies on chicory against HUA have primarily focused on in vivo models and gut microbiota regulation, its direct protective effects and the underlying mechanisms at the cellular level remain less explored. Notably, there is little research utilizing cell metabolomics to explore the metabolic alterations induced by chicory in renal cells. Therefore, this study aimed to systematically investigate the anti-HUA mechanism of chicory by integrating phytochemical and cell-based analyses. To this end, we first characterized the metabolic profiles of chicory root and aerial parts using ultra-high-performance liquid chromatography coupled with quadrupole time-of-flight mass spectrometry (UPLC-QTOF-MS)-based untargeted metabolomics, then employed a non-targeted cell metabolomics strategy coupled with multivariate statistics to identify the potential biomarkers and pathways involved in its protective effect in HK-2 cells.

## 2. Materials and Methods

### 2.1. Chemicals and Reagents

The 21 reference standards were brought from Shanghai Yuanye Bio-Technology Co., Ltd. (Shanghai, China), containing apigenin, apigenin-7-glucuronide, luteolin, luteolin-7-glucoside, luteolin-7-glucuronide, chicoric acid, tartaric acid, caftaric acid, 1,4-dicaffeoylquinic acid, 1,5-dicaffeoylquinic acid, 3,5-dicaffeoylquinic acid, 4,5-dicaffeoylquinic acid, 3-caffeoylquinic acid, 5-caffeoylquinic acid, 4-caffeoylquinic acid, 3-O-(E)-feruloylquinic acid, caffeic acid, lactucin, 8-dexoylactucin, lactupicrin and 11b,13-dihydrolactrcopicrin.

Methanol, acetonitrile, and formic acid were used for analysis from Thermo Fisher Scientific Co., Ltd. (LC-MS grade, Thermo Scientific, Fair Lawn, NJ, USA). Ultrapure water was supplied by a Milli-Q ultrapure water system (Millipore, Billerica, MA, USA). HK-2 (CTCC-002-0018) cells were obtained from Zhejiang Meisen Cell Technology Co., Ltd. (Zhejiang, China). DMEM/F12, FBS, EDTA, and PBS were provided by Beijing Solarbio Science & Technology Co., Ltd. (Beijing, China). Adenosine (A4036-5G) and xanthine oxidase (X4875-10UN, XO) were obtained from Sigma-Aldrich (Sigma-Aldrich, St. Louis, MO, USA). CCK-8 Assay Kit (CK04) was obtained from Dojindo chemical technology Co., Ltd. (Shanghai, China).

### 2.2. Metabolome Between Chicory Roots and Leaves Extracts

#### 2.2.1. Sample Collection and Preparation

A total of 27 root samples of *Cichorium intybus* L. were collected from the Changbai Mountain region in Jilin Province and Xinjiang Province between April and July 2023. Additionally, three batches of *Cichorium intybus* L. aerial parts samples were purchased from Tongrentang Co., Ltd. (Beijing, China) from August to December 2023, which originated in Shandong Province. Chicory applied in our study was identified by Professor Chunsheng Liu from the Medicinal Botany Teaching and Research Section in Beijing University of Chinese Medicine.

The root samples were ground into a fine powder, homogenized, and stored at 4 °C prior to extraction. Approximately 2.0 g of each powdered chicory sample was separately extracted with 20 mL of methanol/water (70:30, *v*/*v*) using an ultrasonic bath (Shumei, KQ5200E, Suzhou, China). Ultrasonication was performed twice for 30 min each, after which the extracts were combined and adjusted to a final volume of 50 mL with the same solvent mixture. The solutions were then centrifuged at 10,000× *g* for 10 min at 4 °C, and the supernatant was filtered through a 0.22 μm nylon membrane for subsequent untargeted LC-MS analysis. To ensure analytical reliability, quality control (QC) samples were prepared by pooling equal volumes of each chicory extract.

#### 2.2.2. UPLC-QTOF-MS Conditions

Chromatographic separation was conducted using a Waters UPLC system (ACQUITY I-Class, Milford, MA, USA) equipped with an ACQUITY BEH T3 column (1.8 μm, 100 mm × 2.1 mm). The column temperature was maintained at 35 °C, and the flow rate was set at 0.3 mL/min. The mobile phase consisted of (A) acetonitrile and (B) water, each containing 0.1% formic acid. A gradient elution program was employed as follows: 0–3 min, 3% A; 3–18 min, 3–40% A; 18–25 min, 40–100% A; 25.1 min, 3% A; 25.1–28 min, 3% A.

Mass spectrometric data were acquired using a Waters Q-TOF/MS system (SYNAPT G2-Si, Milford, MA, USA) equipped with an electrospray ionization (ESI) source operating in both positive and negative ionization modes. Data were collected in MS^E^ continuum mode over a mass range of *m*/*z* 50 to 1200. The following electrospray source parameters were used as follows: capillary voltage: +3.0 kV (ESI+)/−2.5 kV (ESI−), source temperature: 110 °C, desolvation temperature: 450 °C, cone gas flow rate: 50 L/h, desolvation gas flow rate: 900 L/h, and trap collision energy (MS^E^ mode): ramped from 10 to 45 eV. For real-time mass calibration, LockSpray reference ions were infused during acquisition (*m*/*z* 554.2615 for [M−H]^−^ and *m*/*z* 556.2771 for [M+H]^+^).

Quality control (QC) samples, prepared by pooling equal volumes of all experimental samples, were injected every 6 samples to monitor system stability.

#### 2.2.3. Data Processing

Raw data files were processed using Progenesis QI 2.4 (Waters, Milford, MA, USA) for peak extraction, peak alignment, peak area normalization, peak picking, and deconvolution. The peak picking conditions were set based on adduct ion forms as follows: [M+H]^+^, [M+H−H_2_O]^+^, [M+H−2H_2_O]^+^, [M+Na]^+^, [M+K]^+^, [2M+H]^+^, [2M+Na]^+^ for positive ions, and [M−H]^−^, [M−H_2_O−H]^−^, [M+FA−H]^−^, and [2M−H]^−^ for negative ions. Multivariate statistical analysis was performed to compare the metabolic profiles of root and aerial parts extracts. Metabolite identification was then conducted by searching the HMDB database, applying filters for a mass accuracy of <5 ppm, with the highest-scoring results identified by Progenesis QI with a fragmentation similarity score of >0.8. Masslynx 4.1 was used for the chemical analysis with reference standards.

### 2.3. Determination of Anti-Hyperuricemia Activities on HK-2 Cells Induced by Adenosine with Xanthine Oxidase

#### 2.3.1. Sample Preparation for Determination of Anti-Hyperuricemia Activities on HK-2 Cells

Fifty grams of each chicory root and aerial part were separately extracted by refluxing in 500 mL of methanol–water (90:10, *v*/*v*) for 1 h. The resulting extracts were filtered, and the residues were subjected to a second reflux extraction using 500 mL of methanol–water (50:50, *v*/*v*) for an additional hour. The combined filtrates were concentrated using rotary evaporation to remove the solvent completely, yielding 5.4 g of chicory root extract and 8.2 g of chicory aerial parts extract. These extracts were subsequently evaluated for their anti-hyperuricemia effects in HK-2 cells induced by adenosine in the presence of xanthine oxidase.

#### 2.3.2. Cell Culture and Model Construction

HK-2 cells were cultured in DMEM/F12 medium supplemented with 10% fetal bovine serum (FBS) at 37 °C in a humidified 5% CO_2_ atmosphere. Cells were routinely passaged using 0.25% trypsin-0.02% EDTA solution upon reaching 80–90% confluence. A total of 2.5 mmol/L adenosine in serum-free DMEM/F12 and 0.05 U/mL xanthine oxidase (XO) in serum-free DMEM/F12 were prepared, which were sterile-filtered through 0.22 μm membranes, stored at 4 °C, and pre-warmed to 37 °C before use. The study comprised four experimental groups (*n* = 3 per group): control (C): untreated cells, model (M): Adenosine + XO treatment, root extract (CR): adenosine + XO + chicory root extract, aerial parts extract (CL): adenosine + XO + chicory aerial parts extract. Cells were seeded in 6-well plates at a density of 1 × 10^5^ cells/mL (2 mL/well) and maintained with 2 mL medium throughout the experiment. Following 12 h of XO induction, both cell supernatants and pellets were collected for subsequent analysis.

#### 2.3.3. Uric Acid Quantification

Following 12 h of xanthine oxidase (XO) induction, the culture supernatant was collected and centrifuged at 4000 rpm (4 °C, 5 min) to remove insoluble debris. The clarified supernatant was aliquoted and stored at −80 °C until further analysis. To validate the successful establishment of the hyperuricemia (HUA) cell model and assess the anti-hyperuricemia effect of chicory root and aerial parts extracts, uric acid (UA) levels in the supernatant were quantified using a Hitachi 7600 fully automated biochemical analyzer (Shanghai, China).

#### 2.3.4. Statistical Analysis

All data are presented as mean ± standard deviation (SD) from three independent experiments. Statistical analyses were performed using a *t*-test for comparisons with the control group, with a significance threshold set at *p* < 0.05. Data visualization was generated using Origin 8 software (Northampton, MA, USA).

### 2.4. Anti-HUA Mechanism of Chicory Leaves Based on Non-Targeted Cell Metabolomics

#### 2.4.1. Sample Preparation for Non-Targeted Cell Metabolomics

The method was adapted from Pang et al. [18]. Briefly, after collecting the culture supernatant, the cells were digested with trypsin from the 6-well plates and transferred to the centrifuge tubes. The samples were centrifuged at 1000 rpm at 4 °C for 10 min, and the supernatant was discarded. The cells were washed with PBS solution and centrifuged at 1000 rpm at 4 °C for 10 min again, and the supernatant was discarded. A total of 500 μL methanol–water (*v*/*v*, 4:1) was added to the cell samples and ultrasonic for 3 min on ice. The samples were placed in liquid nitrogen for 5 min and frozen at −80 °C for 5 min. This freeze–thaw cycle was repeated three times in total. Subsequently, the samples were centrifuged at 12,000 rpm at 4 °C for 10 min. Finally, the supernatant was collected and stored at −80 °C for metabolomics analysis.

#### 2.4.2. UPLC-Q-TOF-MS Conditions

Chromatography was performed on a UPLC system a Waters UPLC system (ACQUITY I-Class, Milford, MA, USA), equipped with an ACQUITY BEH T3 column (1.8 μm, 100 mm × 2.1 mm). The injection volume was 10.0 μL. The column was kept at 35 °C, and the flow rate was 0.3 mL/min. The mobile phase consisted of 0.1% formic acid in both acetonitrile (A) and water (B). The gradient elution was performed as following protocol: 0–2 min, 5–20% A; 2–12 min, 20–40% A; 12–15 min, 40–70% A; 15–15.1 min, 70–5% A; 15.1–18 min, 5% A.

Mass spectrometry was performed on a Waters Q-TOF/MS instrument (SYNAPT G2-Si, Waters, Milford, MA, USA) with an electrospray ionization source (ESI) in both positive and negative ionization modes, respectively, the same as Section 2.2.2.

#### 2.4.3. Data Acquisition and Processing for Non-Targeted Metabolomics

To equilibrate the system, three QC samples were injected before batch analysis to verify the stability of the instruments, and each QC sample was tested at intervals of six sample injections to assess the stability of the analysis process. The acquired raw data were introduced to Progenesis QI software 2.4 (Waters, Milford, MA, USA) for peak extraction, peak alignment, peak area normalization, peak picking, and deconvolution. All runs were aligned based on an automatically selected QC sample. The peak picking conditions were set based on adduct ion forms as follows: [M+H]^+^, [M+H−H_2_O]^+^, [M+H−2H_2_O]^+^, [M+Na]^+^, [M+K]^+^, [2M+H]^+^, [2M+Na]^+^ for positive ions, and [M−H]^−^, [M−H_2_O−H]^−^, [M+FA−H]^−^, [2M−H]^−^ for negative ions. Metabolites were identified by consulting the HMDB database (http://www.hmdb.ca/, accessed on 8 January 2025). The data matrix in CSV format was established with accurate molecular weight, identification, and peak area, which was introduced to MetaboAnalyst 6.0 (https://www.metaboanalyst.ca/, accessed on 12 January 2025) and SIMCA 14.1 software (Umetrics, Umeå, Sweden) for principal component analysis (PCA) with Pareto scaling mode and orthogonal partial least-squares discriminant analysis (OPLS-DA). A permutation test with 200 cycles was employed, in which R2 and Q2 were usually used to evaluate the quality and reliability of these models. Metabolites were considered significantly different if they had a variable importance in projection (VIP) score greater than 1 and a *p*-value less than 0.05. These differential metabolites were analyzed for pathway enrichment using the KEGG (Kyoto Encyclopedia of Genes and Genomes) database (http://www.genome.jp/kegg/, accessed on 14 January 2025).

## 3. Results

### 3.1. The Untargeted Metabolome and the Characteristic Metabolites Between Chicory Root and Leaf Extracts

**Figure 1:**
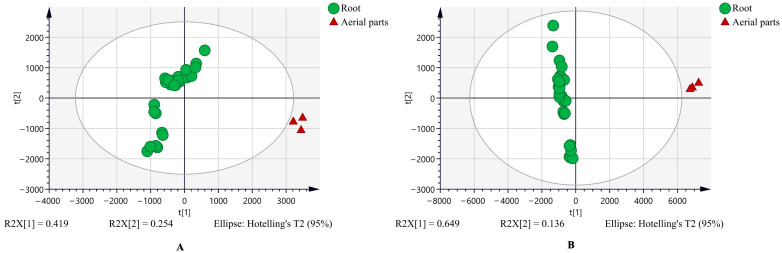
The principal component analysis (PCA) plot between chicory root and aerial parts extracts in the positive mode (**A**) and negative mode (**B**).

A total of 5327 positive ion features and 6260 negative ion features were detected, enabling unsupervised principal component analysis (PCA) to assess overall differences in metabolite profiles. The first two principal components accounted for 58.14% of the total variance in the positive ion mode and 65.04% in the negative ion mode (Figure 1A,B). The PCA score plots indicated clear metabolic distinctions between root and aerial parts extracts.

Characteristic metabolites corresponding to root and aerial parts extracts were identified using the HMDB food database and screened based on variable importance in projection (VIP > 1), *t*-test significance (*p* < 0.05), and fold change (>2). The 52 differential metabolites focused on oligosaccharides, phenolic acids, flavonoids, and sesquiterpenes, some of which were verified with reference standards.

Metabolic profiling showed distinct compositional differences, with phenolic acids and flavonoids being predominantly specific in the aerial parts, whereas sesquiterpenes and oligosaccharides were primarily biosynthesized in the roots. As summarized in Table 1, the root extracts were enriched in sesquiterpenes such as 11b,13-dihydrolactucopicrin, 8-deoxylactucin, lactucin, lactupicrin, cichorioside B, cichorioside F, cichorioside G, cichorioside H, and cichorioside I, while aerial parts extracts contained higher levels of cichorioside J, cichorioside K, and cichoriin. Additionally, chicory roots accumulated substantial amounts of oligosaccharides, including maltooctaose, xyloglucan heptasaccharide, α-L-arabinofuranosyl-(1->3)-β-D-xylopyranosyl-(1->4)-D-xylose, β-D-xylopyranosyl-(1->5)-α-L-arabinofuranosyl-(1->3)-L-arabinose, 3-β-glucosylcellotriose, maltohexaose, cellopentaose, and lactulose. In contrast, cellobiose was more abundant in leaves. This difference likely reflects the high inulin content in chicory roots, a natural storage polysaccharide.

Aerial parts extracts exhibited higher levels of the compounds containing kaempferol derivatives identified as kaempferol-3-glucoside-7-rhamnoside, kaempferol-7-O-neohesperidoside, kaempferol-3-[rhamnopyranosyl-(1->6)-glucoside]-7-rhamnoside, kaempferol-7-sophoroside, and kaempferol-3-arabinofuranoside-7-rhamnofuranoside; apigenin derivatives identified as apigenin, apigenin-7-(6″-O-α-rhamnosyl-β-glucoside), and apigenin-7-glucuronide; luteolin derivatives such as luteolin, luteolin-7-glucoside, luteolin-7-galactoside, luteolin-7-glucuronide, 6-hydroxyluteolin, and luteolin-3′-(3″-acetylglucuronide); anthocyanins such as delphinidin, peonidin-acetyl-3,5-diglucoside, pelargonidin-3-(6″-malonylglucoside), and cyanidin-5-O-β-glucoside; and other flavonoids such as 3,5,7-trihydroxy-4′-methoxy-8-prenylflavone-3-[rhamnosyl-(1->6)-galactoside]-7-galactoside. In contrast, root extracts specifically contained isorhamnetin 3-rutinoside 4′-rhamnoside and kaempferol-3-[glucosyl-(1->3)-rhamnosyl-(1->2)-[rhamnosyl-(1->6)-galactoside], which were absent in aerial parts.

We also found various hydrocinnamic acids containing 3,5-dicaffeoylquinic acid, 4,5-dicaffeoylquinic acid, 3-caffeoylquinic acid, 5-caffeoylquinic acid, 4-caffeoylquinic acid, 3-O-(*E*)-feruloylquinic acid, and caffeic acid in both chicory root and aerial parts extracts. Furthermore, aerial parts extracts were enriched in chicoric acid, tartaric acid, caftaric acid, 2-O-*p*-coumaroyltartronic acid, and 1,5-dicaffeoylquinic acid, and root extracts contained higher levels of 6′-O-trans-caffeoyl-caryoptosidic acid, ferulic acid-4-O-glucuronide, isoferulic acid-3-O-glucuronide, 3-O-caffeoyl-1-O-methylquinic acid, 3-O-(*E*)-feruloylquinic acid, and 4-feruloyl-1,5-quinolactone, as shown in Table 1.

### 3.2. Construction of Hyperuricemia Cell Model

**Figure 2:**
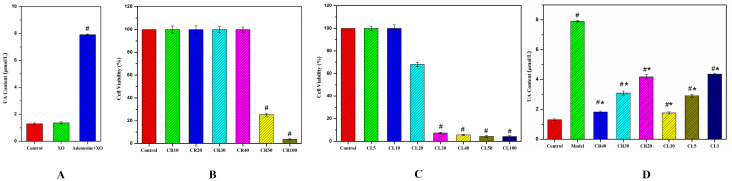
Anti-HUA effect assessment for chicory. (**A**) The levels of UA content in HK-2 cell among the control cells, the cells induced by XO and the cells induced by adenosine with XO; (**B**) CCK-8 assay for chicory root extract of six concentrations at 100 mg/mL (CR100), 50 mg/mL (CR50), 40 mg/mL (CR40), 30 mg/mL (CR30), 20 mg/mL (CR20), and 10 mg/mL (CR10) in the HK-2 cell model; (**C**) CCK-8 assay for chicory aerial parts extract of seven concentrations at 100 mg/mL (CL100), 50 mg/mL (CL50), 40 mg/mL (CL40), 30 mg/mL (CL30), 20 mg/mL (CL20), 10 mg/mL (CL10), and 5 mg/mL (CL5) in the HK-2 cell model; (**D**) the levels of UA for chicory root extracts (CR40, CR30 and CR20) and aerial parts extracts (CL10, CL5, and CL1) in the HK-2 cell model. Data are shown as the mean ± SD (*n* = 3). # *p* < 0.05 vs. control group. * *p* < 0.05 vs. model group.

An adenosine and xanthine oxidase (XO)-induced hypouricemic (HUA) cell model was constructed, which showed a significant increase in uric acid level compared to the control group (Figure 2A). The cytotoxicity of chicory root and aerial parts extracts was evaluated using the CCK-8 assay. Root extracts were tested at concentrations ranging from 100 mg/mL (CR100) to 10 mg/mL (CR10), while aerial parts extracts were assessed from 100 mg/mL (CL100) to 5 mg/mL (CL5). HK-2 cells treated with chicory root extract at concentrations up to 40 mg/mL showed no significant cytotoxicity compared to the control group. A sharp decrease in cell viability was observed at CR50 (25.52 ± 1.26%) and CR100 (3.62 ± 0.64%). In contrast, treatment with chicory aerial parts extract at concentrations up to 10 mg/mL showed no significant cytotoxicity, while a notable reduction in cell viability was observed at CL20 (67.94% ± 1.92) and CL30 (7.39% ± 0.51), as shown in Figure 2B,C. Therefore, the highest non-cytotoxic intervention concentration was determined to be 40 mg/mL for chicory root extract and 10 mg/mL for chicory aerial parts extract.

### 3.3. Anti-Hyperuricemia Effects of Chicory on the UA Levels

In this study, three concentrations separate for chicory root and aerial parts extracts reduced uric acid levels in a concentration- and activity-dependent manner, as demonstrated in Figure 2D. In particular, CR40 and CL10 resulted in notable reductions in UA levels to 1.83 ± 0.07 μmol/L and 1.77 ± 0.07 μmol/L (*p* < 0.05), respectively, compared to the model group (7.91 ± 0.05 μmol/L), bringing them close to the baseline level of the control group (1.30 ± 0.07 μmol/L).

### 3.4. Untargeted Metabolomics Analysis of Cell Metabolome Treated by Chicory Root

#### 3.4.1. Multivariate Statistical Analysis

**Figure 3:**
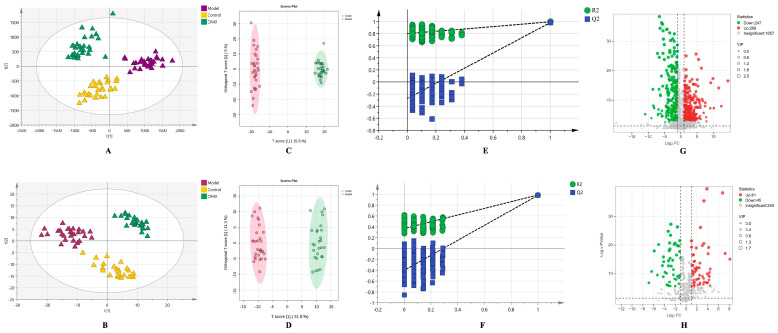
Multivariate statistical analysis of cell metabolites measured by untargeted metabolomics analysis. The score plots of PCA in the positive ion mode (**A**) and the negative ion mode (**B**). Score plot of OPLS-DA between the model group and CR40 group in both the positive ion mode (**C**) and the negative ion mode (**D**). The cross-validation and 200 permutation tests in the positive ion mode (**E**) and the negative ion mode (**F**). The volcano plot in the positive ion mode (**G**) and the negative ion mode (**H**).

Multivariate data analysis is commonly used for evaluating cell metabolome, including fold change analysis, T-test/non-parametric test, principal component (PCA) analysis, partial least squares discrimination (PLS-DA) analysis, orthogonal partial least squares discriminant (OPLSDA) analysis, and so on [19]. PCA selects all recognized variables to exhibit the overall distribution and differences among the cell samples. The position of the QC samples was gathered, and was near the coordinate origin, which confirmed the stability and reproducibility of the analytical method (Figure S1). The PCA score plots displayed obvious separation between the control and the model groups, suggesting that there were evident changes in the cell metabolism of the model group. It was noticeable that the metabolites of the CR40 group were far away from the model group, and closer to the control group at the first principal component, indicating chicory root ameliorates cellular metabolism (Figure 3A,B). Furthermore, OPLS-DA analysis, which can eliminate the noise unrelated to classification information and improve the analytical ability and effectiveness [20], was performed based on the 2893 metabolites recognized in positive ion mode, and the 399 metabolites detected in negative ion mode, respectively. As presented in Figure 3C,D, the score plots of OPLS-DA with R2X = 0.240, R2Y = 0.996 and Q2 = 0.983 in the positive mode as well as R2X = 0.449, R2Y = 0.986 and Q2 = 0.980 in the negative mode, indicated a significant separation between the control group and CR40 group, demonstrating that there were significant differences in metabolite profiles. In addition, as shown in Figure 3E,F, permutation tests were used for validation to avoid overfitting in the OPLS-DA models. The larger values of slope for R2 and Q2 and the small difference (R2 = 0.792, Q2 = −0.274 for positive data, and R2 = 0.371, Q2 = −0.402 for negative data) indicated that there was no overfitting in models and the models were reliable and stable for fitness and prediction [21,22].

#### 3.4.2. Identification of Differential Metabolites Between the Model and CR40 Groups

**Figure 4:**
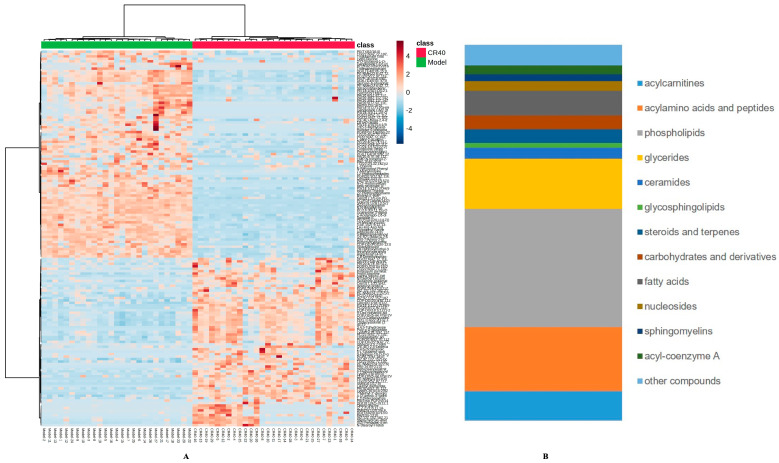
Heatmap of differential metabolites among control group and CR40 group in cluster analysis (**A**) and abundance of differential metabolites with significant differences (**B**).

To explore potential biomarkers of chicory root in regulating uric acid (UA) metabolism, we performed a differential analysis between the model and CR40 groups using univariate and multidimensional statistical methods. The variable importance for the projection (VIP), which is obtained from the OPLS-DA, is usually used to measure the expression patterns of various metabolites and explore biologically significant differential metabolites. Identified metabolites in the positive and negative ion modes with VIP > 1, *p* < 0.01, and |log2FC| > 1 were recognized, in which up-regulated, down-regulated, and non-significant cell metabolites compared with the model group were visualized in volcanic maps, as shown in Figure 3G,H. The screened metabolites that were exposed to environmental and occupational sources or detected as natural products in chicory or other plants were excluded as potential biomarkers. Compared with the model group, the cell samples of the CR40 group had 165 differential metabolites, including 72 up-regulated metabolites and 93 down-regulated biomarkers (Table S2). The hierarchical cluster analysis was performed based on the significantly different metabolites with Pearson as a distance measure, as depicted in Figure 4A. The significantly different metabolites were classified as the compounds of N-acyl amino acid, peptide, acylcarnitine, nucleoside, lipid containing phospholipid and glyceride, and lipid-like molecules (Figure 4B).

First, it was found that *N*-acyl amino acids and peptides containing poly-g-D-glutamate, *N*-palmitoylglycine, *N*-oleoylglycine, *N*-stearoylglycine, N-stearoylhistidine, prolylarginine, Asp-Tyr(SO_3_H)-Met-Gly-Trp-Met-Asp-Phe-NH_2_, Gly-Pro-Gly-Arg-Ala-Phe, and isoleucyl-prolyl-arginine-4-nitroanilide were up-regulated in CR40 group; in addition, *N*-butyrylglycine, phenylpropionylglycine, pentacosanoylglycine, lysylglycine, deoxycholylhistidine, phenylalanylhistidine, histidylleucine, tryptophylserine, histidinyltryptophan, *N*-acetyl-*S*-methylcysteine, *N*-(2-hydroxyethyl)valine, *N*-nervonoyl phenylalanine, *N*,*N*-bis(allyl)-tyr-gly-gly-psi-methylthio-phe-leu, Cys-Tyr-Phe-Gln-Asn-Cys, and Leu-Arg-Asn-Arg were down-regulated in CR40 group.

Secondly, the acylcarnitines of different fatty acyl chains were changed after chicory root intervention. 5-hydroxypentanoylcarnitine, (2*E*,6*Z*)-dodeca-2,6-dienoylcarnitine, 12-hydroxyheptadecanoyl-carnitine, (2*E*)-3-Methylpent-2-enedioyl-carnitine, (2*E*,4*E*)-Hexa-2,4-dienedioylcarnitine, tridecanedioylcarnitine, and (10*E*,15*Z*)-9,12,13-trihydroxyoctadeca-10,15-dienoyl-carnitine were decreased after chicory intervention, while the others such as O-(11-carboxyundecanoyl)carnitine, (5*Z*)-pentadec-5-enoylcarnitine, henicosanoylcarnitine, (9*Z*,12*Z*,15*Z*,18*Z*,21*Z*)-tetracosa-9,12,15,18,21-pentaenoylcarnitine, 3,4,5-trihydroxy-pentanoylcarnitine and 6-hydroxy heptanoylcarnitine were up-regulated.

Thirdly, the levels of lipid molecules were altered by the chicory root, especially glycerol phospholipids and their metabolites. The disturbance of lipid metabolism mainly involves fatty acids (FAs), phosphatidylethanolamine (PE), phosphatidylinositol (PI), phosphatidylcholine (PC), ceramides (Cers), triglycerides (TGs), diacylglycerols (DGs), phosphatidylserines (PSs), lysophosphatidylcholines (LPCs), lysophosphatidylglycerols (LPGs), phosphatidic acids (PAs), sphingomyelins (SMs), phosphatidylglycerophosphates (PGPs), phosphatidylglycerols (PGs), saccharolipids (SLs), and steroids. Notably, volcano plot analysis identified 45 up-regulated and 52 down-regulated differential lipid molecules (Table S2).

#### 3.4.3. Metabolic Pathways Analysis in HUA Cell Model by Chicory Root

**Figure 5:**
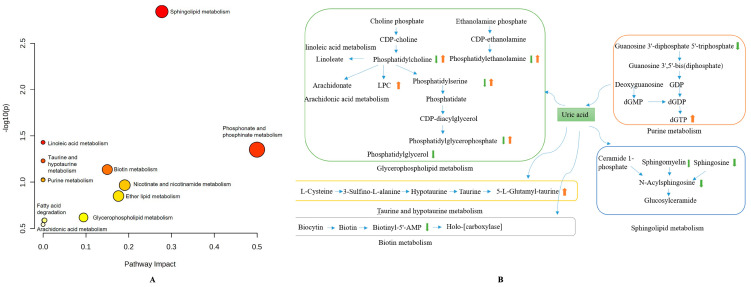
Bioinformatics analysis of differential metabolites. Bubble diagram of differential metabolic pathways for CR40 vs. the control group (The size of the bubble represents the number of genes enriched in each pathway. The color gradient of the bubble, from yellow to orange-red corresponds to the statistical significance of the enrichment, measured by −log10(p), with orange-red hues denoting more significantly enriched pathways) (**A**). The network is based on the CR40 modulation according to the KEGG pathway database. The orange and green arrows indicate up-regulation or down-regulation, respectively, (**B**).

Pathway enrichment analysis was performed on the significantly different metabolites between the model group and CR40 group, using KEGG database. According to the *p*-values from the pathway enrichment analysis and pathway impact values from the pathway topology analysis, chicory root affected HUA cells mainly through the pathways of sphingolipid metabolism, glycerophospholipid metabolism, phosphonate and phosphinate metabolism, linoleic acid metabolism, biotin metabolism, taurine and hypotaurine metabolism, and purine metabolism (Figure 5A). As shown in Figure 5B, the levels of sphingomyelin, sphingosine, *N*-acylsphingosine in sphingolipid metabolism, phosphatidylglycerol in the glycerophospholipid metabolism, biotinyl-5′-AMP in biotin metabolism, guanosine 3′-diphosphate 5′-triphosphatein in purine metabolism in the CR40 group were significantly decreased, while the levels of 5-*L*-Glutamyl-taurine in taurine and hypotaurine metabolism, dGTP in purine metabolism and LPC in the glycerophospholipid metabolism, were increased after the administration with chicory root. We also observed heterogeneous regulations of lipids classified as phosphatidylcholines, phosphatidylethanolamines, phosphatidylserines, and phosphatidylglycerophosphates in the glycerophospholipid metabolism, with both up-regulation and down-regulation in the CR 40 group. It was reported that biotin plays a crucial role in the energy production process of cells by activating carboxylase. It also significantly affects the metabolism of carbohydrates, lipids, and proteins [23]. A study reported that biotin is closely related to renal function and can potentially be a potential metabolic biomarker for urate nephropathy in Uox-Ko mice with a high prediction ability [24]. In our study, we found that bi-otinyl-5′-AMP as the metabolite of biotin was decreased after the treatment by chicory root extract, which is coordinate with the report before.

## 4. Discussions

In our experimental design, the deliberate collection of root samples from diverse geographic regions was intended to capture the natural variation in metabolite profiles that exists in *Cichorium intybus* L. This approach is highly relevant for assessing the quality and authenticity of raw materials in practice, where they are sourced from various habitats. Although the aerial parts samples are limited to three commercial batches, their consistent clustering in the PCA score plot supported the reliability of the data. Despite the observed clustering and separation among root samples in PCA models, which may be related to numerous abiotic and biotic factors, the strong separation between root and aerial parts samples remained the most pronounced. This clearly suggested that organ type is a dominant factor influencing the metabolite profile, overriding the variations caused by environmental factors. In addition, the root and aerial parts samples were not collected from the same individual plants, which introduces an additional source of biological variation. However, the consistent and metabolic differences observed between tissues, which were the primary focus of this investigation, can support our conclusions regarding tissue-specific composition. Future controlled cultivation studies could further refine these findings by eliminating inter-individual variation.

Our analysis indicated that phenolic acids and flavonoids, particularly luteolin derivatives, were predominantly specific in the aerial parts, whereas sesquiterpenes and oligosaccharides were primarily biosynthesized in the root. This finding regarding luteolin derivatives is particularly significant because Yu et al. reported that luteolin alleviates renal injury in hyperuricemic (HN) mice by activating urate excretion and Nrf2/HO-1/NQO1 antioxidant pathways and inhibiting liver xanthine oxidase activity [25]. Phytochemical profiling identified a suite of hydroxycinnamic acids, including 3,5-dicaffeoylquinic acid, 4,5-dicaffeoylquinic acid, multiple mono-caffeoylquinic acids, 3-O-(*E*)-feruloylquinic acid, and caffeic acid in both chicory root and aerial parts, with distinct profiles specific to each organ. This aligns with previous reports on the polyphenolic composition of Cichorium intybus fresh salads, confirmed by the study by Papetti et al. [26]. Notably, several of these compounds, especially the caffeoylquinic acid derivatives we detected, significantly reduce uric acid in hyperuricemic mice by modulating renal transporters (GLUT9, OAT1, URAT1) and inhibiting xanthine oxidase (XO) activity [27]. Furthermore, it was confirmed that chicory root accumulates substantial amounts of oligosaccharides, consistent with its characteristic high inulin content. This finding is pharmacologically relevant, as Guo et al. showed that inulin supplementation alleviates hyperuricemia by upregulating intestinal ABCG2 and downregulating hepatic XOD activity [28]. Collectively, it was suggested that chicory contains multiple bioactive agents with documented anti-hyperuricemic properties, providing a chemical basis for its potential use in treating hyperuricemia.

In the untargeted cell metabolomics, *N*-acyl amino acids and peptides were regulated in CR40 group. *N*-acyl amino acids (NAAAs) are an important family of endogenous signaling molecules in which an amide bond covalently links an amino acid to the acyl moiety of a long-chain fatty acid. NAAAs occur in mammalian tissues and cells at concentrations comparable to other lipid signaling mediators, also emerging as important family of endogenous signaling molecules [29]. Altered levels of *N*-Acyl glycines (NAGlys) in urine have been reported in kidney disease, obesity, and diabetes [30]. *N*-oleoylglycine can activates the peroxisome proliferation proliferator-activated receptor α (PPAR-α), playing a predominant role in the control of energy homeostasis. *N*-palmitoylglycine, *N*-oleoylglycine, and *N*-stereoylglycine can activate the lipid receptor GPR132, one of G-protein-coupled receptors (GPCRs), involved in diverse functions including inflammation and sensing oxidative stress [31]. Nonetheless, future studies are necessary to elucidate the mechanistic roles of *N*-acyl amino acids in the pathogenesis of hyperuricemia. Our preliminary data, showing chicory’s suppression of NF-κB, TNF-α, and IL-1β (Supplementary Figure S3), provide a foundation for these future studies aimed at elucidating the mechanistic roles of *N*-acyl amino acids. It was also indicated that the acylcarnitines of different fatty acyl chains were changed after chicory root intervention. Acylcarnitines are carnitine conjugates used in transporting fatty acids across mitochondria for oxidation [32,33]. The accumulation of acrylcarnitines, especially long-chain species, may indicate lipotoxicity-induced mitochondrial stress. A previous study found that a panel of acylcarnitines was significantly associated with type 2 diabetes, kidney dysfunction, and hyperuricemia [34,35,36]. It was also suggested that lipotoxicity may play a crucial role in the development of insulin resistance and impaired kidney function, which could exacerbate uric acid excretion [34]. Studies suggested that oxidative stress associated with mitochondrial dysfunction might also activate xanthine oxidase to synthesize more uric acid through generating reactive oxygen species [33,34]. Thus, further assays can be conducted for carnitine acetyltransferase activity, to evaluate gene expression of carnitine pathway enzymes. Meanwhile, cellular ATP/GTP levels can be measured to strengthen the anti-HUA mechanistic depth.

Disorders of lipid metabolism existed in the cell model. Abnormal lipid metabolism is characteristic of many diseases, including hyperuricemia. The search for mechanisms behind uric acid-associated cardiovascular risk has identified a promising target in the sphingolipid pathway. Specifically, sphingomyelin synthase 2 (SMS2), critical for converting ceramide to sphingomyelin, has been implicated as a primary sensor of elevated uric acid, translating this metabolic insult into cellular dysfunction. The upregulation of SMS2 by uric acid triggers endoplasmic reticulum (ER) stress, disrupts calcium homeostasis, and ultimately impairs cell survival, migration, and angiogenesis [37]. Recent studies also found that HUA was associated with lipid metabolism pathways, including glycerophospholipid metabolism, sphingolipid metabolism, and glycosylphosphatidylinositol (GPI)-anchor biosynthesis [18,38,39,40]. The findings indicated that CR40 might promote the transformation and metabolism of lipid metabolites and regulate the lipid homeostasis in cell to improve the disorder of lipid metabolism caused by HUA. Hyperuricemia can induce inflammation by promoting proliferation and inflammation, which may lead to an increase in these lipids during hyperuricemia. Some lipids can enhance inflammatory response, destroy mitochondrial integrity and induce apoptosis, where as some lipids like phosphatidylcholine can reduce the oxidative damage of multiple organs and tissues induced by endotoxin, inhibited neutrophil production of various pro-inflammatory mediators [41,42]. By modulating the inflammatory response, CR40 might promote the amelioration of lipid metabolism disorders in hyperuricemia.

## 5. Conclusions

Hyperuricemia is a metabolic disease with increasing incidence rate in recent years. Because of risks for long-term medication intake and many side effects of existing drug treatment, new food therapy with natural food has more advantages. As a traditional medicinal and edible plant in temperate region, *Cichorium intybus* L. is a rich source of bioactive substances for human food fortification. Its anti-HUA effect had been confirmed, but the mechanism still requires in-depth research. This study analyzed and compared the chemical profiles between chicory root and aerial parts using plant metabolomics. A total of 5327 positive ion features and 6260 negative ion features were detected by QTOF, enabling PCA and indicating that there were differences in metabolite profiles. It was found that both chicory root and aerial parts contain various hydrocinnamic acids such as caffeic acid, caffeoylquinic acids, caffeoyltartaric acids, and feruloylquinic acids. Phenolic acids and flavonoids were higher in the aerial parts, whereas sesquiterpenes and oligosaccharides were primarily biosynthesized in the root.

This study also investigated UA levels, potential anti-HUA biomarkers, and pathways of chicory root on HK-2 cells induced by adenosine with xanthine oxidase. The results showed that both chicory root and aerial parts extracts reduce UA levels obviously. By untargeted metabolomics analysis, we found that chicory root influenced the levels of acylcarnitines, acylamino acid, peptides, nucleosides, lipids containing phospholipids, glycerides and other lipid-like molecules in the hyperuricemia cells, and modulated multiple metabolism pathways including sphingolipid metabolism, glycerophospholipid metabolism, phosphonate and phosphinate metabolism, linoleic acid metabolism, biotin metabolism, taurine and hypotaurine metabolism, and purine metabolism to ameliorate the HUA metabolism disorders. Thus, chicory demonstrates beneficial and protective effects against HUA. These findings provide a foundation for future research to elucidate the underlying molecular mechanisms.

## Figures and Tables

**Table 1:** Tentatively identified potential chicory markers in root and leaf extracts from HMDB database using UPLC-Q-TOF-MS.

No.	Tentative Compound Identity	Score ^b^	F Score ^b^	Fold Change ^c^
1	3-galactosyllactose	41.8	25.1	57.16
2	Maltooctaose	47.0	44.8	Infinity ^d^
3	Xyloglucan heptasaccharide	47.0	44.8	Infinity
4	α-L-arabinofuranosyl-(1->3)-β-D-xylopyranosyl-(1->4)-D-xylose	39.9	7.74	113.22
5	β-D-xylopyranosyl-(1->5)- α-L-arabinofuranosyl-(1->3)-L-arabinose	56.0	93.6	36.68
6	3-β-glucosylcellotriose	55.9	84.5	7.91
7	Maltohexaose	51.4	62.4	6.44
8	Cellopentaose	52.8	68.1	9.26
9	Cellobiose	44.0	21.6	311.06
10	Lactulose	41.8	13.4	10.35
11	Kaempferol-3-glucoside-7-rhamnoside	39.6	8.60	64.12
12	Kaempferol-7-O-neohesperidoside	40.0	7.13	27.28
13	Kaempferide-3-[rhamnopyranosyl-(1->6)-glucoside]-7-rhamnoside	44.1	31.8	72.26
14	Kaempferol-7-sophoroside	39.0	8.51	3.56
15	Kaempferol-3-[glucosyl-(1->3)-rhamnosyl-(1->2)-[rhamnosyl-(1->6)-galactoside]]	36.1	0.86	Infinity
16	Apigenin ^a^	40.0	1.41	94.98
17	Apigenin-7-(6″-O-alpha-rhamnosyl-β-glucoside)	37.0	2.53	54.50
18	Apigenin-7-glucuronide ^a^	39.6	4.01	38.34
19	Luteolin ^a^	40.5	6.00	247.53
20	Luteolin-7-glucoside ^a^	38.0	8.46	552.55
21	Luteolin-7-glucuronide ^a^	42.3	23.2	182.87
22	Luteolin-7-galactoside	40.3	6.25	58.45
23	Luteolin-3′-(3″-acetylglucuronide)	36.9	2.27	30.67
24	6-hydroxyluteolin	44.9	33.2	2.18
25	3,5,7-trihydroxy-4′-methoxy-8-prenylflavone-3-[rhamnosyl-(1->6)-galactoside]-7-galactoside	42.9	26.8	8929.80
26	Delphinidin	29.1	8.44	18.38
27	Peonidin-acetyl-3,5-diglucoside	41.9	31.1	Infinity
28	Pelargonidin-3-(6″-malonylglucoside)	39.0	16.7	56,924.90
29	Cyaniding-5-O-β-D-glucoside	35.8	1.91	2115.68
30	Isorhamnetin-3-rutinoside-4′-rhamnoside	39.9	16.1	Infinity
31	Chicoric acid ^a^	42.4	31.8	604,942
32	Tartaric acid ^a^	41.2	9.95	74.82
33	Caftaric acid ^a^	51.7	64.7	29.28
34	2-O-*p*-Coumaroyltartronic acid	52.3	69.8	24.90
35	1,4-dicaffeoylquinic acid ^a^	45.7	49.9	19.00
36	1,5-dicaffeoylquinic acid ^a^	37.2	4.21	6.47
37	6′-O-trans-caffeoyl-caryoptosidic acid	41.1	22.5	11.54
38	Ferulic acid-4-O-glucuronide	49.9	54.2	2.97
39	Isoferulic acid-3-O-glucuronide	43.9	24.2	3.12
40	3-O-caffeoyl-1-O-methylquinic acid	40.1	5.31	12.32
41	3-O-(*E*)-feruloylquinic acid ^a^	41.6	16.0	2.55
42	4-feruloyl-1,5-quinolactone	37.8	2.97	6.52
43	Lactucin ^a^	48.6	48.1	38.00
44	8-deoxylactucin ^a^	44.3	27.6	108.45
45	Lactupicrin ^a^	42.2	30.5	8.06
46	11b,13-dihydrolactucopicrin ^a^	47.4	42.3	99.43
47	Cichorioside B/F/G/H/I	45.2	28.5	8.79
48	Cichorioside B/F/G/H/I	42.4	17.2	40.60
49	Cichorioside B/F/G/H/I	42.0	16.7	6.78
50	Cichorioside J	38.9	4.08	39.49
51	Cichorioside K	37.2	4.71	46.57
52	Cichoriin	45.0	31.9	12.77

^a^ Both retention time and MS/MS spectrum were matched with authentic reference standards; ^b^ the precursor ion match score (score) and the fragment ion match score (F score); ^c^ fold change (FC) was calculated as FC = (mean abundance in roots)/(mean abundance in aerial parts); and ^d^ compounds that were detected in all root samples but were completely absent or below the detection limit in all aerial parts samples.

## Data Availability

The original contributions presented in the study are included in the article; further inquiries can be directed to the corresponding author.

## Supplementary Materials

The following supporting information can be downloaded at: https://www.mdpi.com/article/10.3390/metabo15110727/s1. Figure S1: The deviations of chicory quality control (QC) samples in the principal component analysis (PCA) in positive mode (A) and negative mode (B). Figure S2: The principal component analysis (PCA) score plots of cell metabolomics between samples and QC in positive mode (A) and negative mode (B). Figure S3: Effects of CR40 and CL10 on expression of TNF-α, IL-1β, NF-κB p65, NF-κB p-p65, and NF-κB p-p65/p65 in the HK-2 cell model. Data are shown as the mean ± SD (*n* = 3). # *p* < 0.05 vs. control group and * *p* < 0.05 vs. the model group. Table S1: Tentatively identified potential chicory markers in root and leaf extract from HMDB database using UPLC-Q-TOF-MS. Table S2: Differential metabolites for CR40 group vs. model group with VIP > 1, *p* < 0.01, and |log2FC| > 1.
