# Supplementary material for: Plant Metabolome Between Root and Aerial Parts of Cichorium intybus L. and Anti-Hyperuricemia Mechanisms Based on Cell Metabolomics
Source: Metabolites. 2025 Nov 6;15(11):727. doi: 10.3390/metabo15110727 (PMC12654568; doi:10.3390/metabo15110727)
Supplement: Supplementary file 1 [file metabolites-15-00727-s001.zip › metabolites-3907596-supplementary.pdf]

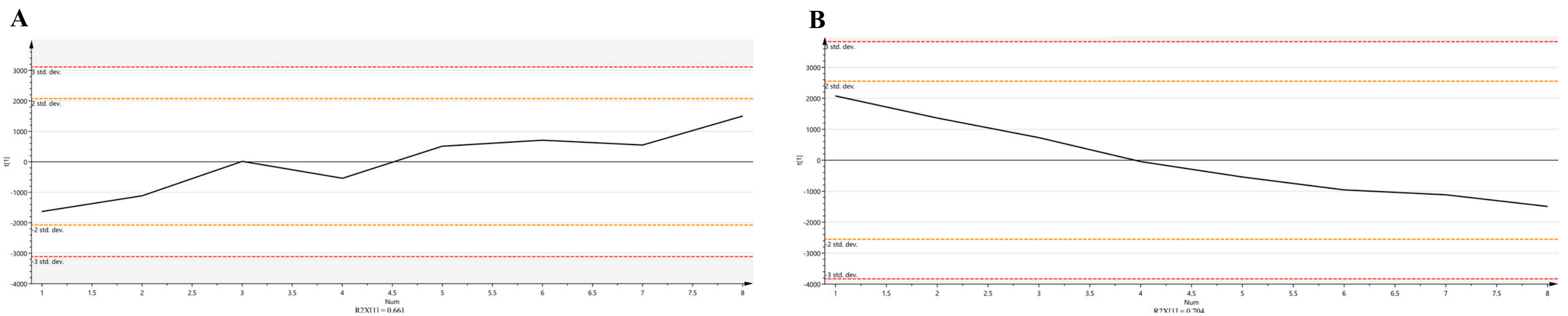

Figure S1 The deviations of chicory quality control (QC) samples in the principal component analysis (PCA) in positive mode (A) and negative mode (B).

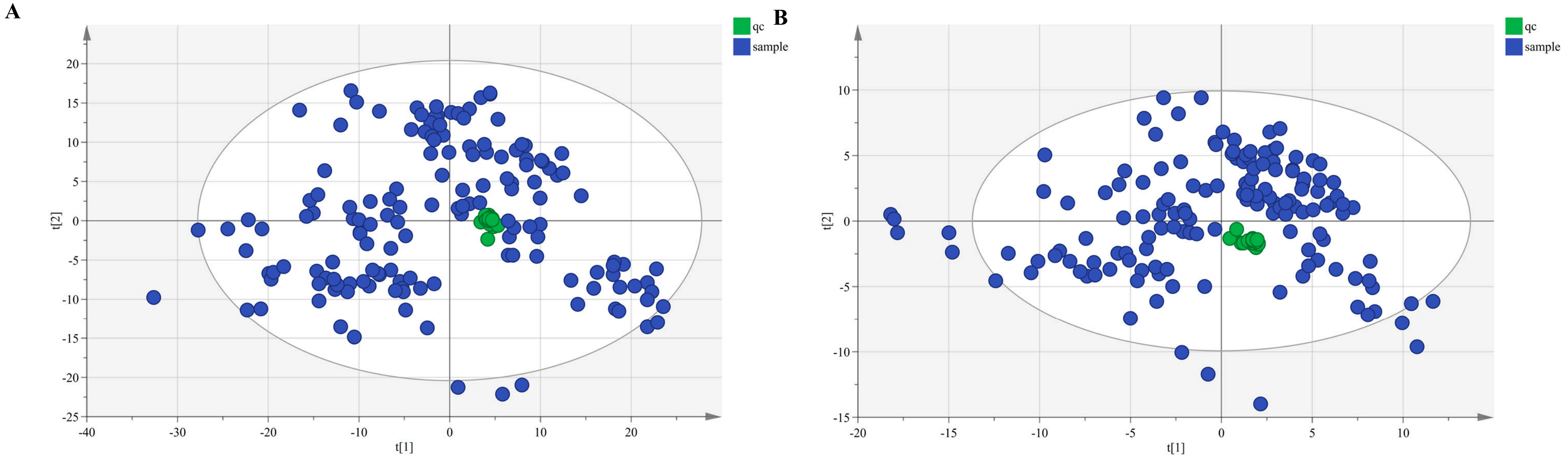

Figure S2 The principal component analysis (PCA) score plots of cell metabolomics between samples and QC in positive mode (A) and negative mode (B).

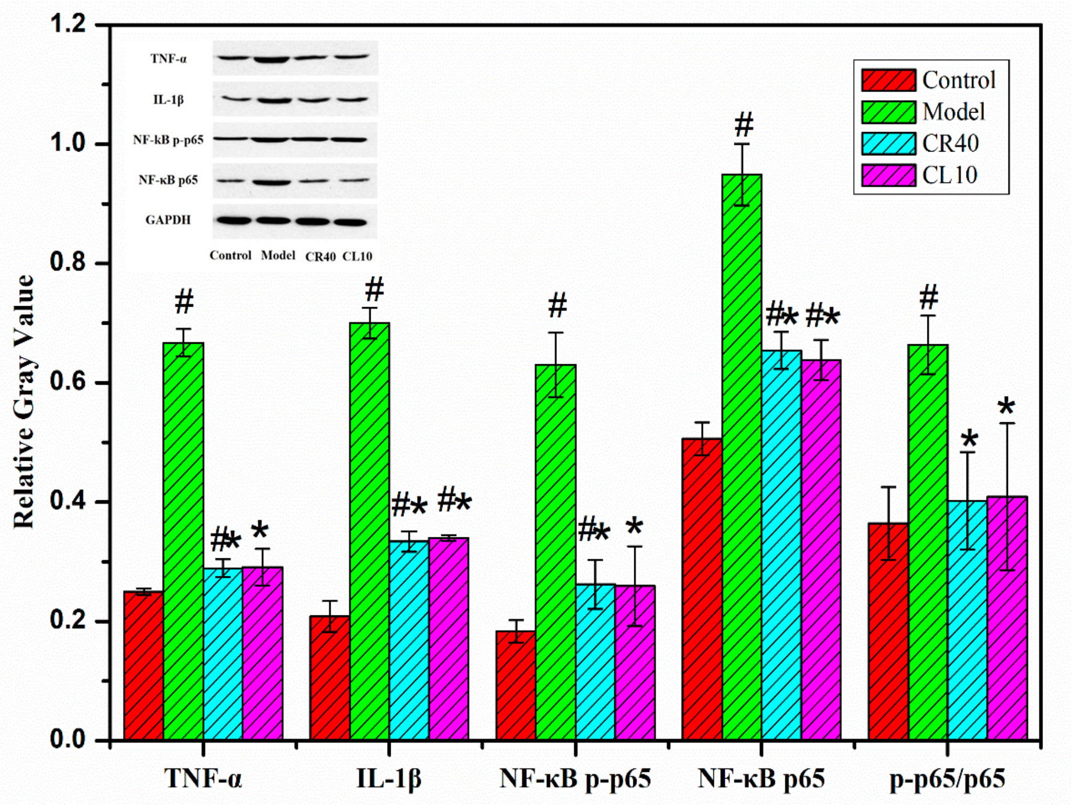

Figure S3 Effects of CR40 and CL10 on expression of TNF-α, IL-1β, NF-κB p65, NF-κB p-p65 and NF-κB p-p65/p65 in HK-2 cell model. Data are shown as the mean ± SD (n = 3). # $p < 0.05$ vs. control group. * $p < 0.05$ vs. model group.

Table S1 Tentatively identified potential chicory markers in root and leaf extract from HMDB database using UPLC-Q-TOF-MS.

No.	*m/z*	Adducts	Formula	Mass Error (ppm)	Score	F Score [a]	Tentative compound identity	Fold Change [c]	Highest Mean	Lowest Mean
1	504.1690	M+FA-H, 2M-H	$C_{18}H_{32}O_{16}$	-0.10	41.8	25.1	3-galactosyllactose	57.16	Root	Aerial parts
2	1313.4273	M-H	$C_{48}H_{82}O_{41}$	1.06	47	44.8	Maltooctaose	Infinity [d]	Root	Aerial parts
3	1061.3414	M-H	$C_{48}H_{82}O_{41}$	1.06	47	44.8	Xyloglucan heptasaccharide	Infinity	Root	Aerial parts
4	459.1355	M+FA-H	$C_{15}H_{26}O_{13}$	-0.10	39.9	7.74	α-*L*-arabinofuranosyl-(1->3)-β-*D*-xylopyranosyl-(1->4)-*D*-xylose	113.22	Root	Aerial parts
5	827.2671	2M-H	$C_{15}H_{26}O_{13}$	-0.39	56	93.6	β-*D*-xylopyranosyl-(1->5)- α-*L*-arabinofuranosyl-(1->3)-*L*-arabinose	36.68	Root	Aerial parts
6	665.2139	M-H	$C_{24}H_{42}O_{21}$	-1.09	55.9	84.5	3-β-glucosylcellotriose	7.91	Root	Aerial parts
7	989.3206	M-H	$C_{36}H_{62}O_{31}$	0.37	51.4	62.4	Maltohexaose	6.44	Root	Aerial parts
8	828.2744	M-H, M+FA-H	$C_{30}H_{52}O_{26}$	-0.36	52.8	68.1	Cellopentaose	9.26	Root	Aerial parts
9	342.1162	M-H, 2M-H, M+FA-H, $M-H_2O-H$	$C_{12}H_{22}O_{11}$	0.10	44	21.6	Cellobiose	311.06	Aerial parts	Root
10	323.0980	$M-H_2O-H$	$C_{12}H_{22}O_{11}$	-1.03	41.8	13.4	Lactulose	10.35	Root	Aerial parts
11	643.2404	M-H	$C_{33}H_{40}O_{13}$	1.17	39.6	8.6	Kaempferol-3-glucoside-7-rhamnoside	64.12	Aerial parts	Root
12	449.1459	M+FA-H	$C_{21}H_{24}O_{8}$	1.49	40	7.13	Kaempferol-7-O-neohesperidoside	27.28	Aerial parts	Root

13	799.2296	M+FA-H	$C_{34}H_{42}O_{19}$	-0.85	44.1	31.8	Kaempferide-3-[rhamnopyranosyl-(1->6)-glucoside]-7-rhamnoside	72.26	Aerial parts	Root
14	609.1457	M-H	$C_{27}H_{30}O_{16}$	-0.61	39	8.51	Kaempferol-7-sophoroside	3.56	Aerial parts	Root
15	903.2802	M+H	$C_{39}H_{50}O_{24}$	4.11	36.1	0.857	Kaempferol-3-[glucosyl-(1->3)-rhamnosyl-(1->2)-[rhamnosyl-(1->6)-galactoside]]	Infinity	Root	Aerial parts
16	269.0454	M-H	$C_{15}H_{10}O_5$	-0.70	40	1.41	Apigenin[b]	94.98	Aerial parts	Root
17	579.1700	M+H	$C_{27}H_{30}O_{14}$	-1.36	37	2.53	Apigenin-7-(6''-*O*-alpha-rhamnosyl-β-glucoside)	54.50	Aerial parts	Root
18	446.0856	M-H, 2M-H	$C_{21}H_{18}O_{11}$	1.58	39.6	4.01	Apigenin-7-glucuronide [b]	38.34	Aerial parts	Root
19	286.0485	M-H, 2M-H	$C_{15}H_{10}O_6$	2.55	40.5	6	Luteolin [b]	247.53	Aerial parts	Root
20	895.1951	2M-H	$C_{21}H_{20}O_{11}$	1.41	38	8.46	Luteolin-7-glucoside [b]	552.55	Aerial parts	Root
21	462.0800	M-H, 2M-H	$C_{21}H_{18}O_{12}$	0.31	42.3	23.2	Luteolin-7-glucuronide [b]	182.87	Aerial parts	Root
22	447.0931	M-H	$C_{21}H_{20}O_{11}$	-0.33	40.3	6.25	Luteolin-7-galactoside	58.45	Aerial parts	Root
23	1050.2181	2M+ACN+H	$C_{23}H_{20}O_{13}$	3.50	36.9	2.27	Luteolin-3'-(3''-acetylglucuronide)	30.67	Aerial parts	Root
24	301.0362	M-H	$C_{15}H_{10}O_7$	2.78	44.9	33.2	6-hydroxyluteolin	2.18	Aerial parts	Root
25	838.2930	M+H-H_2O, M+H	$C_{39}H_{50}O_{20}$	4.09	42.9	26.8	3,5,7-trihydroxy-4'-methoxy-8-prenylflavone-3-[rhamnosyl-(1->6)-galactoside]-7-galactoside	8929.80	Aerial parts	Root
26	284.0321	M-H_2O-H	$C_{15}H_{11}O_7^+$	-1.68	29.1	8.44	Delphinidin	18.38	Aerial parts	Root
27	1333.3714	2M-H	$C_{30}H_{35}O_{17}^+$	2.85	41.9	31.1	Peonidin-acetyl-3,5-diglucoside	Infinity	Aerial parts	Root

28	1083.2268	2M+FA-H	$C_{24}H_{23}O_{13}^{+}$	0.80	39	16.7	Pelargonidin-3-(6"-malonylglucoside)	56924.90	Aerial parts	Root
29	446.0854	M-H_2O-H	$C_{21}H_{21}O_{12}^{+}$	-0.11	35.8	1.91	Cyaniding-5-*O*-β-*D*-glucoside	2115.68	Aerial parts	Root
30	812.2646	M+ACN+H	$C_{34}H_{42}O_{20}$	4.92	39.9	16.1	Isorhamnetin-3-rutinoside-4'-rhamnoside	Infinity	Root	Aerial parts
31	947.1521	2M-H	$C_{22}H_{18}O_{12}$	-0.24	42.4	31.8	Chicoric acid [b]	604942	Aerial parts	Root
32	150.0167	M-H_2O-H, M-H	$C_4H_6O_6$	1.90	41.2	9.95	Tartaric acid [b]	74.82	Aerial parts	Root
33	312.0491	M-H_2O-H, M-H	$C_{13}H_{12}O_9$	3.04	51.7	64.7	Caftaric acid [b]	29.28	Aerial parts	Root
34	311.0415	M-H	$C_{13}H_{12}O_9$	2.07	52.3	69.8	2-O-*p*-Coumaroyltartronic acid	24.90	Aerial parts	Root
35	1031.2462	2M-H	$C_{25}H_{24}O_{12}$	-0.09	45.7	49.9	1,4-dicaffeoylquinic acid [b]	19.00	Aerial parts	Root
36	497.1106	M-H_2O-H	$C_{25}H_{24}O_{12}$	3.28	37.2	4.21	1,5-dicaffeoylquinic acid [b]	6.47	Root	Aerial parts
37	554.1613	M-H, M+FA-H	$C_{25}H_{30}O_{14}$	-4.12	41.1	22.5	6'-O-trans-caffeoyl-caryoptosidic acid	11.54	Root	Aerial parts
38	370.0903	M-H_2O-H, M-H	$C_{16}H_{18}O_{10}$	0.95	49.9	54.2	Ferulic acid-4-O-glucuronide	2.97	Root	Aerial parts
39	351.0724	M+FA-H	C15H14O7	0.78	43.9	24.2	Isoferulic acid-3-O-glucuronide	3.12	Root	Aerial parts
40	349.0929	M-H_2O-H	$C_{17}H_{20}O_9$	0.05	40.1	5.31	3-O-caffeoyl-1-O-methylquinic acid	12.32	Root	Aerial parts
41	367.1038	M-H	$C_{17}H_{20}O_9$	1.06	41.6	16	3-*O*-(*E*)-feruloylquinic acid [b]	2.55	Root	Aerial parts
42	331.0826	M-H_2O -H	$C_{17}H_{18}O_8$	0.68	37.8	2.97	4-feruloyl-1,5-quinolactone	6.52	Root	Aerial parts

43	276.0999	M-H2O-H, M-H	$C_{15}H_{16}O_5$	0.36	48.6	48.1	Lactucin [b]	38.00	Root	Aerial parts
44	241.0872	M-H2O-H	$C_{15}H_{16}O_4$	0.69	44.3	27.6	8-deoxylactucin [b]	108.45	Root	Aerial parts
45	409.1294	M-H	$C_{23}H_{22}O_7$	0.39	42.2	30.5	Lactupicrin [b]	8.06	Root	Aerial parts
46	412.1512	M-H, M+FA-H	$C_{23}H_{24}O_7$	-2.39	47.4	42.3	11b,13-dihydrolactucopicrin [b]	99.43	Root	Aerial parts
47	440.1679	2M-H, M-H, 2M+FA-H, M+FA-H, M-H2O-H	$C_{21}H_{28}O_{10}$	-0.73	45.2	28.5	Cichorioside B/F/G/H/I	8.79	Root	Aerial parts
48	421.1500	M-H2O-H	$C_{21}H_{28}O_{10}$	-0.95	42.4	17.2	Cichorioside B/F/G/H/I	40.60	Root	Aerial parts
49	421.1500	M-H2O-H	$C_{21}H_{28}O_{10}$	-0.98	42	16.7	Cichorioside B/F/G/H/I	6.78	Root	Aerial parts
50	451.1604	M-H	$C_{22}H_{28}O_{10}$	-1.25	38.9	4.08	Cichorioside J	39.49	Aerial parts	Root
51	921.3011	2M+FA-H	$C_{21}H_{26}O_{10}$	-2.58	37.2	4.71	Cichorioside K	46.57	Aerial parts	Root
52	339.0723	M-H	$C_{15}H_{16}O_9$	0.50	45	31.9	Cichoriin	12.77	Aerial parts	Root

[a] Fragmentaion similarity score; [b] matching both retention time and MS/MS spectrum with authentic reference standards; [c] Fold Change (FC) was calculated as FC = (Mean abundance in Roots) / (Mean abundance in Aerial parts); d Compounds that were detected in all root samples but were completely absent (or below the detection limit) in all aerial parts samples.

Table S2 Differential metabolites for CR40 group vs model group with VIP>1, p<0.01 and |log2FC|>1.

No.	Compounds	VIP	p_value [a]	Fold_Change [b]	type	Log2FC
1	SM(d16:1/17:0)	2.275471	1.43E-21	80.251	Up	6.326447
2	PE-NMe(24:0/18:0)	2.184254	4.80E-18	935.59	Up	9.869733
3	PS(18:1(12Z)-2OH(9,10)/18:0)	2.161805	8.48E-18	10.816	Up	3.435095
4	TG(20:5(5Z,8Z,11Z,14Z,17Z)/14:1(9Z)/22:6(4Z,7Z,10Z,13Z,16Z,19Z))	2.128131	3.82E-16	14.546	Up	3.862551
5	Asp-Tyr(SO_3H)-Met-Gly-Trp-Met-Asp-Phe-NH_2	2.059527	9.28E-16	2.3733	Up	1.246894
6	PE(LTE4/22:2(13Z,16Z))	2.075492	1.06E-15	3.7752	Up	1.916553
7	O-(11-Carboxyundecanoyl)carnitine	2.036168	6.11E-15	2.5433	Up	1.346702
8	6alpha,9alpha-Difluoroprednisolone-17-butyrate	2.039755	7.63E-14	7.0296	Up	2.813443
9	dGTP	1.969902	1.00E-13	2.1709	Up	1.118293
10	PC(20:5(7Z,9Z,11E,13E,17Z)-3OH(5,6,15)/20:3(8Z,11Z,14Z))	1.939648	2.50E-12	7.7058	Up	2.945945
11	CDP-DG(5-iso PGF2VI/i-17:0)	1.883068	8.65E-12	2.578	Up	1.366252
12	LysoPC(10:0/0:0)	1.945958	9.06E-12	4.3462	Up	2.119755
13	(5Z)-Pentadec-5-enoylcarnitine	1.91526	1.18E-11	8.8144	Up	3.139862
14	PC(O-14:0/16:0)	1.862481	4.21E-11	2.7819	Up	1.476071
15	PE-NMe(24:1(15Z)/20:3(8Z,11Z,14Z))	1.821633	2.19E-10	2.5114	Up	1.328492
16	Poly-g-D-glutamate	1.765159	5.53E-10	24.477	Up	4.613355
17	Lauroyl diethanolamide	1.780601	6.01E-10	24.653	Up	4.623691
18	PA(i-24:0/i-21:0)	1.709435	5.85E-09	62.268	Up	5.960419
19	9-Oxo-nonanoic acid	1.675985	6.55E-09	2.4647	Up	1.301412
20	CDP-DG(20:4(8Z,11Z,14Z,17Z)-2OH(5S,6R)/i-18:0)	1.659824	1.3E-08	2.0433	Up	1.030901
21	Henicosanoylcarnitine	1.690317	1.38E-08	9.985	Up	3.319762
22	Prolyl-Arginine	1.665859	1.88E-08	3.1736	Up	1.66612

23	1,2-Didecanoyl-sn-glycero-3-phosphocholine	1.648899	2.21E-08	3.1982	Up	1.67726
24	5-L-Glutamyl-taurine	1.672465	2.95E-08	33.587	Up	5.069831
25	(6Z,9Z,12Z,15Z,18Z)-3-Oxotetracosapenta-6,9,12,15,18-enoyl-CoA	1.594821	1.32E-07	2.8765	Up	1.524314
26	CDP-DG(22:6(4Z,7Z,11E,13Z,15E,19Z)-2OH(10S,17)/i-21:0)	1.552698	1.42E-07	2.3607	Up	1.239215
27	LysoPG(18:2(9Z,12Z)/0:0)	1.501465	3.73E-07	2.6869	Up	1.425943
28	Cer(d17:1/22:5(4Z,7Z,10Z,13Z,19Z)-O(16,17))	1.55379	4.63E-07	16.151	Up	4.013552
29	PC(22:5(4Z,7Z,10Z,13Z,19Z)-O(16,17)/2:0)	1.421308	1.63E-06	3.1284	Up	1.645425
30	(9Z,12Z,15Z,18Z,21Z)-Tetracosa-9,12,15,18,21-pentaenoylcarnitine	1.438184	4.52E-06	11.672	Up	3.54498
31	Palmitoyl glycine	1.366975	1.44E-05	11.753	Up	3.554957
32	Glutamine-glutamate	1.329235	1.46E-05	2.1573	Up	1.109227
33	PA(8:0/i-13:0)	1.349147	1.50E-05	19.902	Up	4.314842
34	PGP(LTE4/20:2(11Z,14Z))	1.31317	2.27E-05	3.2022	Up	1.679063
35	DG(18:1(9Z)-O(12,13)/0:0/i-17:0)	1.334986	3.84E-05	16.165	Up	4.014802
36	Potassium palmitate	1.242546	7.31E-05	6.0914	Up	2.606774
37	9-Hydroxy-10-O-D-glucuronoside-12Z-octadecenoate	1.250134	0.000081	8.045	Up	3.008092
38	DG(14:0/0:0/18:3(9,11,15)-OH(13))	1.255036	0.000101	6.2706	Up	2.648603
39	3beta-hydroxy-4beta-methyl-5alpha-cholest-7-ene-4alpha-carboxylate	1.244718	0.000109	4.5964	Up	2.200504
40	PE-NMe2(24:0/22:6(4Z,7Z,10Z,13Z,16Z,19Z))	1.208529	0.000126	51.4	Up	5.683696
41	Oleoyl glycine	1.206727	0.000156	19.771	Up	4.305314
42	DG(TXB2/0:0/i-13:0)	1.186492	0.000181	11.822	Up	3.563402
43	CDP-DG(5-iso PGF2VI/i-22:0)	1.18455	0.000223	2.9147	Up	1.543347
44	DG(22:6(4Z,7Z,11E,13Z,15E,19Z)-2OH(10S,17)/0:0/i-20:0)	1.138178	0.000266	3.3249	Up	1.733311
45	(Z,Z,Z)-1,8,11,14-Heptadecatetraene	1.169493	0.00028	10.297	Up	3.364152
46	2,4,6-Octatriynoic acid	1.173706	0.000391	4.976	Up	2.314986
47	TG(22:6(4Z,7Z,10Z,13Z,16Z,19Z)/22:5(7Z,10Z,13Z,16Z,19Z)/O-18:0)	1.100729	0.000504	2.6157	Up	1.387197

48	Gly-Pro-Gly-Arg-Ala-Phe	1.086505	0.000533	3.7567	Up	1.909466
49	Undeca-3,5,7-trienoyl-CoA	1.103464	0.000735	5.2542	Up	2.393471
50	(2E,4E)-2,4-Dodecadienal	1.091924	0.000774	2.0478	Up	1.034075
51	Stearoylglycine	1.072007	0.000806	9.6188	Up	3.265857
52	N-Stearoyl Histidine	1.106122	0.000818	7.2012	Up	2.848237
53	SM(d19:1/20:3(6,8,11)-OH(5))	1.069756	0.001093	8.5002	Up	3.087497
54	PE(20:4(7E,9E,11Z,13E)-3OH(5S,6R,15S)/DiMe(13,5))	1.008099	0.001343	2.0639	Up	1.045373
55	PA(5-iso PGF2VI/14:1(9Z))	1.041226	0.001465	4.8539	Up	2.279144
56	PGP(LTE4/i-18:0)	1.038151	0.001658	4.3666	Up	2.12651
57	(9Z,12Z,15Z,18Z,21Z)-Tetracosa-9,12,15,18,21-pentaenoyl-CoA	1.011097	0.001734	4.652	Up	2.217851
58	DG(PGJ2/0:0/i-18:0)	1.032582	0.002032	6.1915	Up	2.630289
59	SM(d19:1/24:1(15Z))	2.463121	4.89E-39	0.010561	Down	-6.56511
60	Guanosine 3'-diphosphate 5'-triphosphate	2.444415	1.08E-34	0.048987	Down	-4.35146
61	PC(PGF1alpha/14:0)	2.439892	3.26E-34	0.022674	Down	-5.46282
62	N-Butyrylglycine	2.423562	1.66E-31	0.18066	Down	-2.46865
63	PC(18:3(9,11,15)-OH(13)/14:1(9Z))	2.423896	1.98E-31	0.024691	Down	-5.33987
64	PE(PGD1/20:1(11Z))	2.43485	1.04E-30	0.24302	Down	-2.04085
65	N,N-Bis(allyl)-tyr-gly-gly-psi-methylthio-phe-leu	2.392984	1.40E-29	0.02936	Down	-5.09
66	5-Hydroxypentanoylcarnitine	2.43661	2.05E-29	0.48378	Down	-1.04758
67	Cys-Tyr-Phe-Gln-Asn-Cys	2.417392	1.67E-28	0.24737	Down	-2.01526
68	Deoxycholylhistidine	2.372886	1.51E-27	0.096204	Down	-3.37776
69	N-Acetyl-S-methylcysteine	2.386	2.68E-27	0.2567	Down	-1.96184
70	Tryptophyl-Serine	2.385309	1.36E-26	0.022579	Down	-5.46887
71	Histidinyl-Tryptophan	2.372036	1.49E-26	0.007029	Down	-7.15242
72	Sphingosine	2.343196	2.96E-25	0.010295	Down	-6.60191

73	Spirolide E	2.298363	1.43E-23	0.42314	Down	-1.24079
74	PS(18:1(12Z)-2OH(9,10)/15:0)	2.333672	1.63E-23	0.37167	Down	-1.42791
75	2-Acetamido-2,6-dideoxygalactose	2.251996	5.24E-22	0.43995	Down	-1.18459
76	(2E,6Z)-Dodeca-2,6-dienoylcarnitine	2.305798	3.55E-21	0.35617	Down	-1.48936
77	22-Hydroxydocosanoic acid	2.250871	9.04E-20	0.060734	Down	-4.04135
78	24-Methylcycloart-23-en-3beta-yl acetate	2.200328	6.47E-19	0.016308	Down	-5.93828
79	PE(18:1(9Z)-O(12,13)/16:0)	2.211575	1.23E-18	0.23219	Down	-2.10662
80	Phenylalanylhistidine	2.238527	2.05E-18	0.14635	Down	-2.77251
81	PE(P-16:0/18:4(6Z,9Z,12Z,15Z))	2.202924	7.45E-18	0.082495	Down	-3.59955
82	(2s)-7-Amino-2-{[(R)-Hydroxy{(1r)-2-Methyl-1-[(3-Phenylpropanoyl)amino]propyl}phosphoryl]methyl}heptanoic Acid	2.221338	1.11E-17	0.25555	Down	-1.96832
83	Biotinyl-5'-AMP	2.202606	1.62E-17	0.091017	Down	-3.45772
84	Cer(d17:1/5-iso PGF2VI)	2.152406	6.47E-17	0.27607	Down	-1.85689
85	2,8-Dimethylnonan-5-one	2.136435	1.20E-16	0.2173	Down	-2.20224
86	DG(LTE4/0:0/i-17:0)	2.157342	1.24E-16	0.37158	Down	-1.42826
87	PE-NMe(22:6(4Z,7Z,10Z,13Z,16Z,19Z)/22:6(4Z,7Z,10Z,13Z,16Z,19Z))	2.15292	1.73E-16	0.36613	Down	-1.44957
88	Cer(t18:0/TXB2)	2.131004	5.60E-16	0.14929	Down	-2.74381
89	PC(20:5(7Z,9Z,11E,13E,17Z)-3OH(5,6,15)/P-16:0)	2.106209	2.17E-15	0.30193	Down	-1.72771
90	DG(LTE4/0:0/i-15:0)	2.098642	5.79E-15	0.25392	Down	-1.97755
91	PE-NMe(22:6(4Z,7Z,10Z,13Z,16Z,19Z)/18:3(9Z,12Z,15Z))	2.084098	6.80E-15	0.15462	Down	-2.6932
92	Dimethyltryptamine	2.083776	1.00E-14	0.045543	Down	-4.45663
93	PA(20:4(5Z,8Z,11Z,14Z)-OH(17)/18:3(9Z,12Z,15Z))	2.086589	1.97E-14	0.009765	Down	-6.67812
94	PE(20:4(5Z,7E,11Z,14Z)-OH(9)/22:5(7Z,10Z,13Z,16Z,19Z))	2.05293	2.58E-14	0.33311	Down	-1.58593
95	Cer(d18:1/25:0)	2.004348	8.71E-14	0.29825	Down	-1.74541
96	PGP(20:5(7Z,9Z,11E,13E,17Z)-3OH(5,6,15)/a-25:0)	2.01074	1.27E-13	0.059365	Down	-4.07424

97	Tetracosahexaenic acid	2.012132	1.63E-13	0.33519	Down	-1.57695
98	N-(2-Hydroxyethyl)valine	1.956528	1.39E-12	0.012864	Down	-6.28052
99	Phenylpropionylglycine	1.935051	2.66E-12	0.073127	Down	-3.77345
100	beta-nicotinate D-ribonucleotide	1.915469	3.28E-12	0.013765	Down	-6.18285
101	Histidylleucine	1.9618	5.49E-12	0.39948	Down	-1.3238
102	CDP-DG(PGD2/i-12:0)	1.890829	1.37E-10	0.14276	Down	-2.80834
103	PA(PGD1/24:1(15Z))	1.844492	1.84E-10	0.091682	Down	-3.44722
104	Pentacosanoylglycine	1.727032	2.33E-09	0.37533	Down	-1.41377
105	TG(22:6(4Z,7Z,10Z,13Z,16Z,19Z)/18:4(6Z,9Z,12Z,15Z)/O-18:0)	1.751094	3.34E-09	0.46519	Down	-1.10411
106	PC(20:5(6E,8Z,11Z,14Z,17Z)-OH(5)/17:0)	1.721292	6.57E-09	0.41471	Down	-1.26983
107	PE(14:1(9Z)/5-iso PGF2VI)	1.716914	7.73E-09	0.002965	Down	-8.3977
108	DG(22:5(4Z,7Z,10Z,13Z,19Z)-O(16,17)/0:0/i-17:0)	1.698582	1.83E-08	0.04825	Down	-4.37333
109	Ganglioside GM2 (d18:0/20:0)	1.671113	4.24E-08	0.003446	Down	-8.18107
110	TG(22:6(4Z,7Z,10Z,13Z,16Z,19Z)/20:4(8Z,11Z,14Z,17Z)/22:6(4Z,7Z,10Z,13Z,16Z,19Z))	1.635074	4.59E-08	0.32617	Down	-1.6163
111	PGP(PGF1alpha/i-22:0)	1.623626	6.06E-08	0.069408	Down	-3.84875
112	N-Nervonoyl Phenylalanine	1.564214	8.27E-08	0.13358	Down	-2.90422
113	PS(18:3(9,11,15)-OH(13)/14:1(9Z))	1.621389	1.31E-07	0.008682	Down	-6.84777
114	PI(22:5(4Z,7Z,10Z,13Z,19Z)-O(16,17)/22:2(13Z,16Z))	1.555157	3.79E-07	0.017978	Down	-5.79762
115	Creatinine citrate	1.584784	4.12E-07	0.20182	Down	-2.30886
116	12-Hydroxyheptadecanoylcarnitine	1.479569	4.54E-07	0.04907	Down	-4.34901
117	PA(20:3(8Z,11Z,14Z)/20:1(11Z))	1.51926	9.51E-07	0.30255	Down	-1.72475
118	1-alkyl-2-acylglycerophosphoethanolamine	1.514689	1.74E-06	0.3268	Down	-1.61352
119	TG(14:0/20:4(5Z,8Z,11Z,14Z)/20:0)	1.421756	2.06E-06	0.045208	Down	-4.46728
120	PA(PGJ2/i-16:0)	1.479718	3.63E-06	0.013817	Down	-6.17741

121	PA(18:1(12Z)-2OH(9,10)/18:4(6Z,9Z,12Z,15Z))	1.45559	4.78E-06	0.007912	Down	-6.9818
122	Glucose-6-phosphate lactate	1.428458	1.35E-05	0.15325	Down	-2.70604
123	fructose-6-phosphate pyruvate	1.394243	1.66E-05	0.068859	Down	-3.86021
124	(2E)-3-Methylpent-2-enedioylcarnitine	1.357132	1.71E-05	0.042942	Down	-4.54147
125	Ganglioside GD3 (d18:0/22:0)	1.382269	0.000019	0.019672	Down	-5.66771
126	PA(20:5(7Z,9Z,11E,13E,17Z)-3OH(5,6,15)/P-16:0)	1.347345	2.87E-05	0.020245	Down	-5.62629
127	PG(20:4(5Z,7E,11Z,14Z)-OH(9)/i-12:0)	1.345962	3.33E-05	0.017231	Down	-5.85885
128	TG(22:2(13Z,16Z)/22:1(13Z)/O-18:0)	1.284627	3.77E-05	0.26084	Down	-1.93876
129	Triglutamate folate	1.274738	4.64E-05	0.24334	Down	-2.03895
130	18-oxo-oleate	1.279783	6.53E-05	0.22755	Down	-2.13574
131	CMP-N-trimethyl-2-aminoethylphosphonate	1.241977	7.39E-05	0.21459	Down	-2.22035
132	PS(16:1(9Z)/14:1(9Z))	1.279662	7.45E-05	0.0435	Down	-4.52284
133	PA(LTE4/i-20:0)	1.245027	7.51E-05	0.22207	Down	-2.17091
134	(2E,4E)-Hexa-2,4-dienedioylcarnitine	1.288515	0.000125	0.49239	Down	-1.02213
135	L-Coprine	1.184224	0.000159	0.34216	Down	-1.54726
136	2-O-Glutaroyl-1-O-palmitoyl-sn-glycero-3-phosphocholine	1.198192	0.000172	0.37307	Down	-1.42248
137	PA(20:4(7E,9E,11Z,13E)-3OH(5S,6R,15S)/10:0)	1.130276	0.000331	0.42888	Down	-1.22135
138	PG(LTE4/18:3(9Z,12Z,15Z))	1.110703	0.000775	0.32727	Down	-1.61145
139	Lysyl-Glycine	1.096439	0.000791	0.42892	Down	-1.22122
140	PA(20:3(8Z,11Z,14Z)-2OH(5,6)/i-22:0)	1.074556	0.001005	0.4494	Down	-1.15393
141	Tridecanedioylcarnitine	1.029153	0.002183	0.28989	Down	-1.78642
142	PC(5-iso PGF2VI/16:1(9Z))	1.04562	0.002613	0.035791	Down	-4.80426
143	DHBOA-Glc	1.569783	3.04E-21	9.7498	Up	3.285373
144	7-Methyl-2'-deoxyguanosine-3'-monophosphate	1.534602	1.26E-17	16.963	Up	4.084319
145	Cholylaspartic acid	1.485558	3.46E-17	2.3758	Up	1.248413

146	PGP(20:4(5Z,7E,11Z,14Z)-OH(9)/i-14:0)	1.413132	2.48E-14	5.8687	Up	2.553041
147	LysoPC(0:0/18:2(9Z,12Z))	1.374294	2.90E-12	22.154	Up	4.469495
148	Cer(d20:1/18:1(12Z)-2OH(9,10))	1.358535	2.93E-12	3.8975	Up	1.962549
149	4-(2-amino-3-hydroxyphenyl)-4-oxobutanoic acid O-glucoside	1.301232	3.05E-11	3.1116	Up	1.637657
150	3,4,5-Trihydroxypentanoylcarnitine	1.285462	4.48E-11	20.18	Up	4.334854
151	TG(22:6(4Z,7Z,10Z,13Z,16Z,19Z)/15:0/O-18:0)	1.316128	5.82E-11	2.3482	Up	1.231555
152	(6Z,9Z,12Z,15Z)-octadeca-6,9,12,15-tetraenoyl-CoA	1.335037	2.36E-10	2.5171	Up	1.331763
153	6-Hydroxyheptanoylcarnitine	1.292197	3.98E-10	5.6186	Up	2.490211
154	CDP-DG(18:1(12Z)-2OH(9,10)/i-12:0)	1.257809	5.24E-10	18.165	Up	4.183089
155	PA(20:4(5Z,7E,11Z,14Z)-OH(9)/i-12:0)	1.222898	1.40E-08	3.8018	Up	1.926683
156	Isoleucyl-prolyl-arginine-4-nitroanilide	1.049798	8.11E-07	5.8638	Up	2.551836
157	2R-Hydroxymethyl-3S-hydroxypyrolidine	1.649796	3.95E-24	0.13576	Down	-2.88087
158	N-Eicosapentaenoyl aspartic acid	1.61795	7.26E-21	0.029509	Down	-5.0827
159	(10E,15Z)-9,12,13-Trihydroxyoctadeca-10,15-dienoylcarnitine	1.54149	2.13E-18	0.43259	Down	-1.20893
160	Leu-Arg-Asn-Arg	1.517208	3.43E-17	0.29557	Down	-1.75843
161	tetrahydrogeranylgeranyl-PP	1.523425	4.97E-16	0.12714	Down	-2.97551
162	Argipressin (4-9)	1.52241	2.26E-15	0.18163	Down	-2.46093
163	PC(24:1(15Z)/22:2(13Z,16Z))	1.198123	1.44E-08	0.28075	Down	-1.83264
164	PE(TXB2/16:0)	1.115987	4.62E-07	0.3976	Down	-1.33061
165	7-Methylinosine	1.02286	2.63E-06	0.13231	Down	-2.91801

[a] Data were analyzed by an unpaired, two-sided Student's t-test (CR40 group *vs*. model group); [b] The Fold Change (FC) was calculated as FC = (Mean abundance in CR40 group) / (Mean abundance in the model group)
